# Chromosome-scale scaffold genome sequence of *Arthrobacter citreus* strain NJF-O2 isolated from the distal colon of a healthy sheep

**DOI:** 10.1128/mra.00200-26

**Published:** 2026-04-30

**Authors:** A. S. Wadday, Baraa Akeel Al-Hasan, Ali H. D. Janabi

**Affiliations:** 1 Department of Anesthesia Techniques, College of Medical Technology, The Islamic University, Najaf, Iraq; 2 Iraqi Biosafety Association, Najaf, Iraq; 3 Department of Veterinary Microbiology, College of Veterinary Medicine, University of Al-Qadisiyah 362928, Al-Diwaniyah, Iraq; 4 Department of Medical Laboratory Technology, College of Medical Technology, The Islamic University, Najaf, Iraq; 5 Department of Laboratory, Al Najaf Veterinary Hospital, Najaf, Iraq; University of Maryland School of Medicine, Baltimore, Maryland, USA

**Keywords:** *Arthrobacter citreus*, chromosome-scale scaffold genome, ruminant microbiota

## Abstract

*Arthrobacter citreus* strain NJF-O2 was isolated from the distal colon of a healthy adult ram postmortem in Najaf Governorate, Iraq. We present the chromosome-scale scaffold genome sequence of this strain to expand *Arthrobacter* genomic resources and enable high-resolution comparative and functional genomic studies of its metabolic plasticity and environmental adaptability.

## ANNOUNCEMENT

*Arthrobacter* species are gram-positive, high-guanine-cytosine (G + C) Actinobacteria commonly found in soil, rhizospheres, and other environments renowned for their metabolic diversity, stress tolerance, and bioremediation potential (1). The genus *Arthrobacter* has undergone substantial taxonomic revision based on phylogenetic and genomic evidence, and chromosome-scale scaffold genome sequences provide stable references for comparative analyses within the genus (2). This chromosome-scale scaffold expands genomic resources for *Arthrobacter* and provides a useful reference for comparative analyses of genome architecture, gene context, and ecological diversity within the genus. *Arthrobacter citreus* strain NJF-O2 was isolated from the distal colon of a clinically healthy adult ram obtained postmortem at a licensed slaughterhouse in Najaf Governorate, Iraq. Approximately 1 g of luminal content and multiple mucosal swabs were collected using sterile swabs, placed in sterile containers on ice, and transported promptly to the laboratory. Upon receipt, aliquots were prepared as 10-fold serial dilutions in sterile phosphate-buffered saline and plated on Brucella blood agar, clarified rumen fluid agar, and brain heart infusion agar (Thermo Fisher Scientific, USA). Plates were incubated at 37°C for 48 h in a candle jar under reduced-oxygen conditions (~3–5% O_₂_). For whole-genome sequencing, a loopful of purified culture was applied to an ANICARD (SAN Group Biotech Germany GmbH) and shipped under sterile conditions to SAN Group Laboratories (Germany) for DNA extraction and quantification. Genomic DNA was extracted from a pure culture using a commercial kit. DNA quality and purity were assessed spectrophotometrically (UV-1900i Plus, Shimadzu), and samples with A260/280 ratios of 1.8–2.0 were used for sequencing, and sequencing libraries were prepared using the Watchmaker DNA Library Prep Kit (Watchmaker Genomics) and sequenced on the Illumina MiSeq platform (2 × 151 bp paired-end reads), yielding approximately 3.3 million read pairs and an estimated genome coverage of 184×. Reads were quality-trimmed using Trimmomatic v0.39 (3) and *de novo* assembled with SPAdes v3.15.3 (4). Assembly statistics calculated with QUAST v5.2.0 (5) showed that the initial assembly comprised 14 contigs, with an N50 of 875,329 bp and a largest contig of 1,101,369 bp. GTDB-Tk v2.4.0, data release r220 (6), classified the genome as Arthrobacter_B citreus based on average nucleotide identity (ANI) screening against reference genome GCF_009192745.1 (ANI, 98.97%; alignment fraction, 0.958). The draft contigs were first scaffolded with MeDuSa v1.6 (7) using multiple closely related *Arthrobacter* reference genomes (Table S1; available at https://doi.org/10.6084/m9.figshare.31796188), yielding two scaffolds. The resulting assembly was then refined with RagTag v2.1.0 (8) using the closely related reference genome GCF_029023805.1 (*A. citreus* strain DSM 15046), producing a chromosome-scale scaffold. Genome completeness and contamination were assessed using CheckM v1.2.3 (9), which estimated the assembly to be 98.39% completeness with 0.98% contamination. The scaffold was reoriented to begin at the dnaA gene using Circlator’s fixstart module (10). Assembly consistency was assessed by comparing terminal scaffold regions using SeqKit v2.6.1 (11) and NUCmer (MUMmer v4.0.0) (12). Default parameters were used for all software, unless otherwise noted. Because the final assembly structure was informed by reference-guided scaffolding, the genome is conservatively described here as a chromosome-scale scaffold. The chromosome-scale scaffold comprises 3,527,331 bp with a G + C content of 65.63%. Annotation via the National Center for Biotechnology Information (NCBI) Prokaryotic Genome Annotation Pipeline (PGAP v6.10) (13) predicted 3,300 genes, 3,239 protein-coding sequences, 52 tRNA genes, one complete rRNA operon (5S, 16S, 23S), four noncoding RNAs, and 28 pseudogenes. This chromosome-scale scaffold genome sequence of *A. citreus* strain NJF-O2 provides a useful genomic resource for future comparative and functional studies.

## Data Availability

The chromosome-scale scaffold genome sequence of *Arthrobacter citreus* strain NJF-O2 has been deposited in GenBank under accession CP197463. The associated BioProject accession number is PRJNA1251031, and the BioSample accession number is SAMN47990684. Raw sequencing reads have been deposited in the Sequence Read Archive under accession SRR36990657.
